# Augmentation of the Ulnar Motor Nerve Repair with Anterior Interosseous Nerve in High Ulnar Nerve Palsy: Our Clinical Experience

**DOI:** 10.1055/s-0044-1801805

**Published:** 2025-02-03

**Authors:** Gopika Jith, Kaushik Mahadik, Santanu Suba, Sanjay Kumar Giri

**Affiliations:** 1 Department of Burns and Plastic Surgery, All India Institute of Medical Sciences, Bhubaneswar, Orissa, India

**Keywords:** anterior interosseous nerve, high ulnar nerve, proximal ulnar nerve palsy

## Abstract

Following proximal ulnar nerve repair, there will be a delay in innervating the distally placed intrinsic muscles of the hand, which can lead to irreversible damage to the intrinsic motor end plates. Supercharging with end-to-side anterior interosseous nerve (AIN) transfer can augment the results of nerve repair by babysitting the motor end plates and thus preventing its denervation. Recently, there have been discussions regarding whether AIN, which contains only 500 axons, can augment the ulnar motor branch, which contains approximately 1,500 axons. In one of our cases, electromyogram following surgery showed activity in the first dorsal interossei and abductor digiti minimi without any signs of reinnervation in the flexor carpi ulnaris. This may support the contribution of AIN in supplying the intrinsic muscles. Considering the low morbidity of the procedure and potential improvements in muscle strength, all patients undergoing ulnar nerve repair in high ulnar nerve palsies can be counseled to undergo an end-to-side AIN transfer.

## Introduction

High ulnar nerve palsies result in restricted hand functions. With proximal ulnar nerve repair alone, the time required to reinnervate the distal intrinsic muscles of the hand can lead to irreversible damage to the intrinsic motor end plates. If supercharging can be performed, providing end-to-side anterior interosseous nerve (AIN) transfer, we will be able to prevent denervation and maintain the number of motor end plates. Unlike end-to-end AIN transfer, this surgery will not interfere with proximal nerve innervation and will serve to augment functional recovery. Recently, there have been discussions regarding whether the AIN, which contains only 500 axons, can augment the ulnar motor branch, which contains approximately 1,500 axons supplying 14 intrinsic muscles. In contrast, in one of our cases, 18 months following surgery, the first dorsal interossei and abductor digiti minimi showed activity in electromyogram (EMG) without any signs of reinnervation in the flexor carpi ulnaris. This may support the contribution of AIN in supplying the intrinsic muscles.

## Case Report

Two young male patients (aged 21 and 23 years) presented with a history of traumatic cut injury to the ulnar nerve. The patients presented 4 months (case 1) and 7 months (case 2) after their injuries. Both the cases had high ulnar nerve palsy. They had no history of any interventions done for the same. The patients did not have any comorbidities or any significant family histories. Both cases presented with complaints of weakness and clawing of the hand, as well as absent sensory perception over the ulnar aspect.

**Fig. 1:**
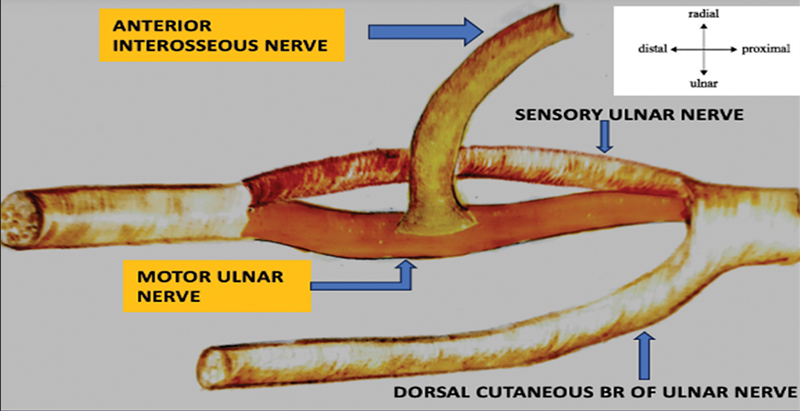
Anterior interosseous nerve (AIN) sutured to the epineurium of the ulnar nerve motor fascicular group.

**Fig. 2:**
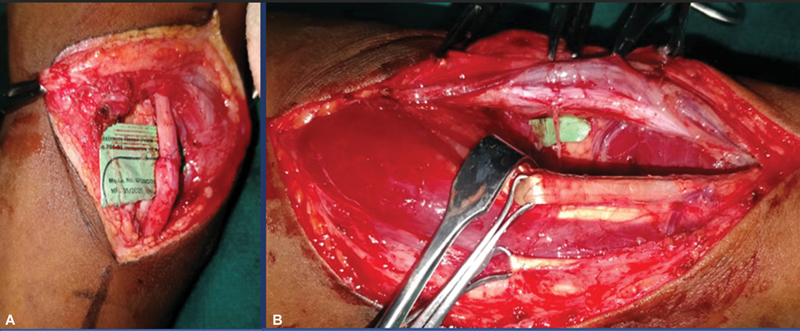
( **A** ) Proximal repair of the ulnar nerve. ( **B** ) Distal forearm: anterior interosseous nerve (AIN) to ulnar nerve end-to-side coaptation.

Under tourniquet, primary ulnar nerve repair was done proximally. An incision was made ulnar to the thenar crease, extending 9 to 10 cm proximal to the wrist crease. Guyon's canal was decompressed, and ulnar nerve neurolysis was performed. Motor ulnar nerve was identified based on the internal topography. The AIN was dissected from its entry to the pronator quadratus muscle and transected distally. A perineurial window was created over the motor ulnar nerve. The AIN was sutured to the epineurium of the ulnar motor fascicular group using 9–0 nylon simple interrupted sutures in a tension-free manner ( Figs. 1 and 2 ).

Muscle strengths of the first dorsal interosseous, abductor digiti minimi, and adductor pollicis were assessed and recorded according to the Medical Research Council grading system. Grip strength was assessed using q Jamar dynamometer, and pinch strength was assessed using a pinch gauge. Both were compared with the normal contralateral side ( Table 1 ). Preoperative EMG was performed to establish a baseline of nerve and muscle function and postoperative EMG assessments were conducted at regular 6-month intervals to monitor the recovery.

**Table 1:** MRC/grip strength/pinch strength recording of patients 1 and 2

Time	MRC		Grip strength (% of normal)		Pinch strength (% of normal)	
Patients	1	2	1	2	1	2
Pre-op	M0	M0	–	–	–	–
6 mo	M3	M3	20	11	40	50
12 mo	M3	M3	33	45	60	80
18 mo	M3	M3	35	65	80	80

Abbreviation: MRC, Medical Research Council.

Clawing deformity was not corrected in both cases and showed only minimal improvement in the proximal interphalangeal joint extension lag. Forearm pronation in both cases was assessed during follow-up and was found to be intact.

## Discussion

High ulnar nerve injuries result in poor recovery of the hand function. In the past, end-to-end AIN transfers were done to the motor branch of the ulnar nerve, which prevented innervation from the native ulnar nerve. 1 2 In cases of end-to-side nerve transfers, innervation from the proximal native nerve is augmented with the AIN. This procedure, which was first reported by Barbour et al, has a protective effect similar to the babysitting procedure for cross-facial nerve grafting. 3 In a systemic review, 100% of patients who underwent this procedure showed intrinsic function recovery in an average time of 3.6 months. 4 Although intrinsic function recovery has been shown in the literature, in a study by Arami and Bertelli, no improvement was seen regarding claw deformity. 5 In our cases, only a slight improvement in clawing was observed. In conjunction with AIN transfer, techniques to restore sensation to the ulnar border of the hand showing good results have been described. 4 6

There is a disparity in the number of axons of the AIN supplying pronator quadrates and the ulnar motor branch supplying 14 intrinsic muscles of the hand. The ratio of the axon numbers of the AIN to those of the ulnar motor branch is 1:2 to 1:4. 1 7 8 There are around 1,500 axons transmitting signals from the deep branch of the ulnar nerve, whereas only around 500 axons are present in the terminal branch of the AIN. 9 10 The distance to be covered from the site of coaptation, which could be 10 to 20 cm, is also questionable because regeneration will take around 100 to 200 days. 10 However, EMG of one of our cases revealed activity in the first dorsal interossei and abductor digiti minimi without any signs of reinnervation in the flexor carpi ulnaris, favoring the contribution by the AIN, although more evidence is required to draw a conclusion.

No complications were encountered in our cases. The pronator quadratus is a forearm pronator but is expendable if the pronator teres is intact. Both of our cases had no difficulty in pronation during follow-up. Although this procedure of augmenting the proximal ulnar nerve repair with AIN end-to-side coaptation has been found to have variable outcomes in previous studies, morbidity is low. Both of our patients had good ulnar intrinsic motor recovery. Because of the procedure's low morbidity and potential improvements in hand function, all patients with proximal ulnar nerve injuries can be counseled to undergo an end-to-side AIN to ulnar motor nerve transfer.

## Conclusion

- Distal AIN transfer can augment proximal ulnar nerve repair.
- The efficacy of the procedure has been questioned in the recent past.
- No major complications were associated with the procedure.
- EMG evidence showed activity through AIN innervation.
- Patients undergoing proximal ulnar nerve repair can be advised to undergo AIN transfer.
